# The use of photobiomodulation therapy for the prevention of chemotherapy-induced peripheral neuropathy: a randomized, placebo-controlled pilot trial (NEUROLASER trial)

**DOI:** 10.1007/s00520-022-06975-x

**Published:** 2022-03-21

**Authors:** Lodewijckx Joy, Robijns Jolien, Claes Marithé, Evens Stijn, Swinnen Laura, Lenders Hilde, Bortels Sandra, Nassen Wendy, Hilkens Ruth, Raymakers Liesbeth, Snoekx Sylvana, Hermans Sylvia, Mebis Jeroen

**Affiliations:** 1 Faculty of Medicine and Life Sciences, Hasselt University, Martelarenlaan 42, 3500 Hasselt, Belgium; 2 Department of Medical Oncology, Jessa Hospital, Stadsomvaart 11, 3500 Hasselt, Belgium; 3 Department of Neurology, Jessa Hospital, Stadsomvaart 11, 3500 Hasselt, Belgium

**Keywords:** Chemotherapy, Peripheral neuropathy, Polyneuropathy, Photobiomodulation, Breast cancer

## Abstract

**Purpose:**

The purpose of this study was to investigate the effectiveness of photobiomodulation (PBM) therapy for the prevention of chemotherapy-induced peripheral neuropathy (CIPN) in breast cancer patients.

**Methods:**

A prospective, randomized placebo-controlled pilot trial (NEUROLASER) was set up with 32 breast cancer patients who underwent chemotherapy (ClinicalTrials.gov; NCT03391271). Patients were randomized to receive PBM (*n* = 16) or placebo treatments (*n* = 16) (2 × /week) during their chemotherapy. The modified Total Neuropathy Score (mTNS), six-minute walk test (6MWT), Numeric pain Rating Scale (NRS), and Functional Assessment of Cancer Therapy/Gynecologic Oncology Group Taxane (FACT/GOG-Taxane) were used to evaluate the severity of CIPN and the patients’ quality of life (QoL). Outcome measures were collected at the first chemotherapy session, 6 weeks after initiation of chemotherapy, at the final chemotherapy session, and 3 weeks after the end of chemotherapy (follow-up).

**Results:**

The mTNS score increased significantly over time in both the control and the PBM group. A significantly higher score for FACT/GOG-Taxane was observed in the PBM group during chemotherapy compared to the control group. Questions of the FACT/GOG-Taxane related to sensory peripheral neuropathy symptoms showed a significant increase in severeness over time in the control group, whereas they remained constant in the PBM group. At follow-up, a (borderline) significant difference was observed between both groups for the 6MWT and patients’ pain level, in benefit of the PBM group.

**Conclusions:**

This NEUROLASER trial shows promising results concerning the prevention of CIPN with PBM in breast cancer patients. Furthermore, a better QoL was observed when treated with PBM.

**Supplementary Information:**

The online version contains supplementary material available at 10.1007/s00520-022-06975-x.

## Introduction

Due to better cancer therapies, cancer survival rates have improved over the last decades. Unfortunately, the incidence of long-term treatment-related side effects and chronic toxicities, such as chemotherapy-induced peripheral neuropathy (CIPN), also increased [1]. The sensory nervous system is mainly affected during CIPN, resulting in pain, allodynia, tingling, and numbness in the hands and feet. Though, motor and/or autonomic dysfunction can also occur [2]. CIPN can significantly impact the patient’s general health, resulting in increased health care costs and a diminished quality of life (QoL) [3–5]. Furthermore, treatment outcomes can adversely be affected by forcing a chemotherapy dose reduction or premature treatment discontinuation [6].

The meta-analysis of Seretny et al*.* demonstrated that the overall incidence of CIPN lies around 68% in the first month after chemotherapy. After 3 months, an incidence of 60% was observed, and at 6 months or more, it was reduced to 30% [7]. However, the incidence varies according to the chemotherapeutic agent used, the duration of exposure, and the dose. Platinum-based drugs, taxanes, vinca alkaloids, and bortezomib are known to cause the highest incidence of CIPN [7].

The molecular mechanism inducing neuropathy, axonopathy, and/or myelinopathy, which contributes to the pathogenesis of CIPN, is largely unknown. Multiple targets of the peripheral nervous system are affected, including changes in axonal transport, mitochondrial dysfunction, oxidative stress, and loss of intraepidermal nerve fibers. Moreover, alterations can occur in multiple ion channels, neurotransmitters, and their receptors’ expression levels. However, the pathogenesis of CIPN highly depends on the chemotherapeutic agent used, but a mechanistic basis remains unclear [8].

The available evidence for preventive and therapeutic options for CIPN is limited. Pharmacological treatment with tricyclic antidepressants and anticonvulsants is minimally effective and not well tolerated [9–12]. Currently, the American Society of Clinical Oncology (ASCO) only recommends duloxetine to treat CIPN [2, 13].

A new, emerging, and preventive therapy in the supportive care of cancer patients is photobiomodulation (PBM) therapy. PBM uses a visible and/or (near)-infrared light produced by laser diodes or light-emitting diodes. In general, PBM will increase cell viability by activating the cytochrome c oxidase of the electron transport chain, resulting in increased mitochondrial respiration and increased activity of molecules such as adenosine triphosphate (ATP), nitric oxide (NO), reactive oxygen species (ROS), calcium ions, and various other signaling molecules [14–17].

In vivo and in vitro studies have shown that PBM accelerates and enhances axonal growth and regeneration, and suppresses neural apoptosis [18–21]. Several clinical trials on PBM and diabetic neuropathy demonstrated beneficial effects [22–25]. Moreover, one animal study and two clinical trials showed improvement in CIPN symptoms when PBM was applied [26–29]. However, these studies focused on the therapeutic application of PBM instead of its preventive use. Therefore, this trial aimed to investigate whether PBM can prevent the development of CIPN in breast cancer patients.

## Methods

### Study design

A prospective, randomized placebo-controlled pilot trial (NEUROLASER trial) evaluated the effectiveness of PBM in the prevention of CIPN in breast cancer patients. Patients were divided into a control group, receiving placebo treatments, or a PBM group, receiving PBM. All patients received taxane-based chemotherapy at the Limburg Oncology Center (LOC, Jessa Hospital, Hasselt, Belgium) between December 2017 and June 2021. Both the ethics committees of the Jessa Hospital and the University of Hasselt approved the study (B243201733877). The study was registered at ClinicalTrials.gov (NCT03391271).

### Study population

Patients were eligible for inclusion if they were diagnosed with invasive (stages 1, 2, and 3A) breast adenocarcinoma, aged 18 years or above, and were planned to undergo at least three cycles of 3-weekly docetaxel (100 mg/m^2^) or nine cycles of weekly paclitaxel (80 mg/m^2^). Exclusion criteria were a history of neuropathy before the start of the trial due to other medical conditions, usage of stable doses of medication to treat peripheral neuropathy (e.g., duloxetine and pregabalin), metastatic disease, interruption of the chemotherapy for more than two cycles, interruption of more than two consecutive PBM sessions, and reduction of the chemotherapy dosage. Patients were recruited at the oncology department of the Jessa Hospital (Hasselt, Belgium) 1 week before the start of the taxane treatment. Written informed consent was obtained before the start of the study.

### Randomization

Eligible patients were randomized (1:1) into a control group or PBM group. Patients were allocated based on a block randomization process, with a block size of four using a computer-generated random number list. Only the PBM operator knew the allocation of the patients in the groups. Patients were blindfolded to ensure that they did not know in which group they were allocated. After the follow-up visit, patients were informed whether they were allocated to the PBM or placebo group.

### Intervention

#### Chemotherapy

Breast cancer patients were first treated with a combination of epirubicin (100 mg/m^2^) and cyclophosphamide (600 mg/m^2^) for four cycles, every 3 weeks, followed by a 3-weekly administration of docetaxel (100 mg/m^2^) or a weekly administration of paclitaxel (80 mg/m^2^) whether or not in combination with carboplatin (AUC of 5 mg/ml).

#### Photobiomodulation

Patients in the PBM group received PBM twice weekly during their taxane treatment (12–18 weeks, depending on the chemotherapy regime). A trained operator provided PBM using a class IV MLS M6 laser (ASA Srl, Vicenza, Italy). This device combines two laser diodes of two different wavelengths (905 and 808 nm), peak power (25 W and 1.1 W), and emission mode (pulsed and continuous). The two laser beams work simultaneously and synchronously with coincident propagation axes. A power density of 0.168 W/cm^2^ and a fluence of 4 J/cm^2^ were used. Patients were treated bilaterally at the upper limbs (nervus medialis, ulnaris, and radialis), the back (L4-S1), and lower limbs (sciatic nerve, plantar, and dorsal surface of the feet). The beam spot size ranges from 3 to 19.625 cm^2^, depending on the treatment zone. More specific PBM parameters can be found in the supplementary Table 1. During the placebo treatments, the PBM device did not emit light. All patients, independently of their treatment group, wore safety glasses during treatment to prevent eye damage.

### Outcome measures

Data were collected at the start of taxane treatment, 6 weeks after initiation of taxane treatment, at the end of taxane treatment, and 3 weeks after the end of taxane treatment (follow-up visit).

#### Patient data

Patient’s personal, disease-, and treatment-related characteristics were collected via patient questionnaires and the patient’s medical records to rule out possible risk factors for developing CIPN.

#### Peripheral neuropathy

The validated modified Total Neuropathy Score (mTNS) was defined as the primary endpoint to determine whether or not the patients developed CIPN. The mTNS assesses six domains of sensory and motor neuropathy. Scores range from 0 to 24, with higher scores indicating a more severe grade of neuropathy [30]. In addition, the patient’s aerobic capacity and endurance was investigated by performing the six-minute walk test (6MWT), according to the standard protocol. The 6MWT measures the distance an individual is able to walk over a total of six min on a hard, flat surface and is adjusted for sex, age, and BMI [31]. A numeric rating scale (NRS) ranging from 0 (no pain) to 10 (worst pain imaginable) was used to assess the patients’ pain level.

#### Quality of life

The patient’s QoL was assessed by using the validated Functional Assessment of Cancer Therapy/Gynecologic Oncology Group Taxane (FACT/GOG-Taxane) questionnaire [32]. Questions from the different subscales (physical well-being, social/family well-being, emotional well-being, functional well-being, and additional concerns specific for taxane-treated patients) were rated according to the 5-point Likert Scale. A higher score indicates a better QoL.

### Statistical analysis

SPSS Statistics 26.0 (IBM, Chicago, IL) was used to perform statistical analysis. Patients and therapy-related characteristics were analyzed by performing a Student’s *t* test, Fisher’s exact test, and Pearson Chi-square test, as appropriate. Primary and secondary endpoints were analyzed by mixed analysis of variances (ANOVA), Friedman test, and Mann–Whitney *U* test, as appropriate. The level of significance was set assuming a significance level of 5% (*P* < 0.05, two-tailed).

## Results

**Fig. 1:**
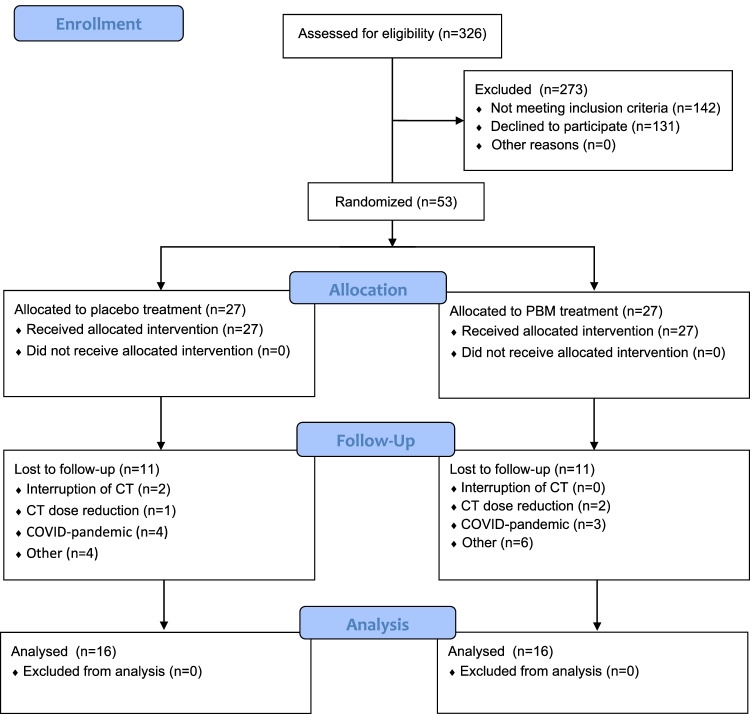
Flowchart. PBM, photobiomodulation; CT, chemotherapy

A total of 323 breast cancer patients were assessed on eligibility between December 2017 and January 2021. Twenty-seven patients were randomized to the control group and 27 patients to the PBM group. In total, 22 patients were lost for follow-up, with as main reason the COVID-19 pandemic, an interruption or alteration in the chemotherapy regimen, and other reasons such as claustrophobia and low adherence to the study protocol. Analysis was performed on 32 patients, with 16 patients in each group (Fig. 1). There were no significant differences between the demographical, disease- and treatment-related data between the two groups, except for exercise frequency (Table 1).

**Table 1:** Patient characteristics

	Control group (*n* = 16)		PBM group (*n* = 16)		
**Demographics**	Mean ± SD		Mean ± SD		*P*^a^
Age	49.75 (11.25)		51.88 (11.31)		0.85
BMI	25.14 (3.24)		25.20 (4.78)		0.12
	*n*	%	*n*	%	*P*^b^
Smoking habits					0.34
Current	2	12.50	0	0.00	
Former	6	37.50	7	43.75	
Never	8	50.00	9	56.25	
Alcohol consumption					0.28
Never or < 1 unit a week	9	56.25	5	31.25	
1–3 units a week	2	12.50	5	31.25	
4–10 units a week	4	25.00	6	37.50	
11–20 units a week	1	6.25	0	0.00	
Exercise frequency					0.05*
Never	5	31.25	0	0.00	
Once a week	2	12.50	5	31.25	
2–3 times a week	4	25.00	8	50.00	
3–4 times a week	4	25.00	1	6.25	
≥ 5 times a week	1	6.25	2	12.50	
**Disease-related**					
Tumor location					1.00
Left	9	56.25	10	62.50	
Right	7	43.75	6	37.50	
Tumor type					0.23
Invasive ductal adenocarcinoma	13	81.25	16	100.00	
Invasive lobular adenocarcinoma	3	18.75	0	0.00	
T-stage					0.61
1	3	18.7562.50	5	31.25	
2	10		10	62.50	
3	2	12.50	1	6.25	
4	1	6.25	0	0.00	
N-stage					0.66
0	7	43.75	7	43.75	
1	6	37.50	8	50.00	
2	2	12.50	1	6.25	
3	1	5.25	0	0.00	
Prognostic factors					
Estrogen positive	13	81.25	11	68.75	0.69
Progesterone positive	9	56.25	12	75.00	0.46
Excess HER2 protein	3	18.75	8	50.00	0.14
Triple negative	3	18.75	3	18.75	1.00
**Chemotherapy-related**					
Exposure					0.33
Paclitaxel	8	50.00	7	43.75	
Paclitaxel and carboplatin	1	6.25	4	25.00	
Docetaxel	7	43.75	5	31.25	
Timing					0.29
Neoadjuvant	5	31.25	9	56.25	
Adjuvant	11	68.75	7	43.75	

*BMI*, body mass index; *PBM*, photobiomodulation; *SD*, standard deviation

* was set assuming a significance level of 5%

^a^Student’s *t* test (two-tailed)

^b^Chi-squared tests (two-tailed), or Fisher’s exact tests, as appropriate (two-tailed)

### Primary endpoint

**Fig. 2:**
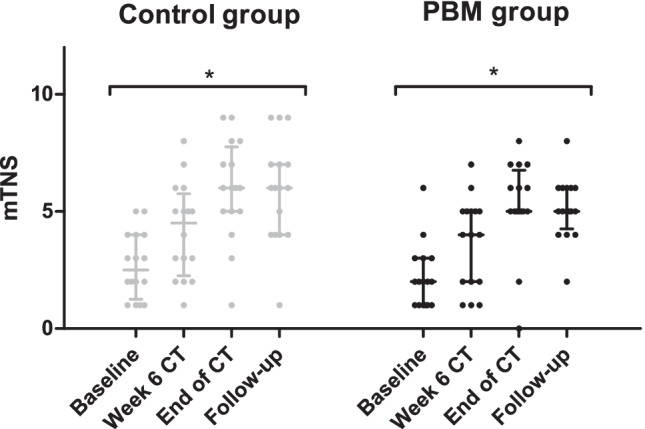
Modified Total Neuropathy Score (mTNS). Comparison of mTNS between the control group (*n* = 16) and the PBM group (*n* = 16) over time. Data are presented as median ± interquartile range. A higher score indicates a more severe grade of peripheral neuropathy. For both groups, a significant increase of mTNS was observed (*P*s < 0.001) using the Friedman test. PBM, photobiomodulation; CT, chemotherapy

The primary endpoint of this trial was a difference in mTNS over time between both groups. A significant increase of the mTNS over time was observed in the control and the PBM group (*P*s < 0.001). Although not significant, a visual analysis showed a trend in which the control group appears to have a higher mTNS at the end of chemotherapy and follow-up than the PBM group (Fig. 2).

### Secondary outcomes

**Fig. 3:**
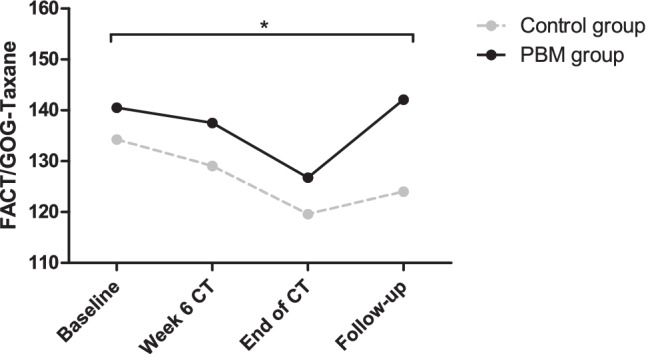
Functional Assessment of Cancer Therapy/Gynecologic Oncology Group Taxane (FACT/GOG-Taxane) total score. Comparison of FACT/GOG-Taxane total score between the control group (*n* = 15) and the PBM group (*n* = 16) over time. Data are shown as means and a higher score indicates a better quality of life (QoL). Mixed ANOVA revealed significant main time effect, and time by group interaction (*P*s < 0.036). No significant main group effect was observed (*P* = 0.067). PBM, photobiomodulation; CT, chemotherapy

Figure 3 demonstrates the progression of the QoL of the patients during the trial. Data of only 31 patients, control group (*n* = 15) and PBM group *(n* = 16), was included for this analysis since one patient did not return the questionnaire at follow-up. There was a significant main time effect and time by group interaction for the FACT/GOG-Taxane total score (*P*s < 0.036). No significant main group effect was observed (*P* = 0.067). In addition, the subscales physical well-being, emotional well-being, and functional well-being of the FACT/GOG-Taxane showed a significant difference at follow-up between the control group and the PBM group (*P*s < 0.040). However, also a significant difference was observed in the subscale functional well-being at baseline (*P* = 0.044); thus, true significance at follow-up is questioned. Although the total neurotoxicity subscale did not show any significant differences, questions related to sensory peripheral neuropathy symptoms (NTX1, NTX2, NTX4, NTX9) demonstrated a significant increase in severeness over time in the control group (*P*s < 0.010), whereas they remained constant in the PBM group (Supplementary Fig. 2–3).

**Fig. 4:**
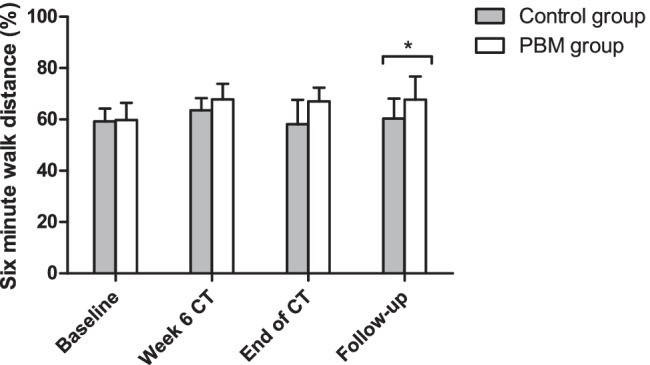
Six-minute walk test (6MWT). Comparison of percent predicted 6MWT distance between the control group (*n* = 16) and the PBM group (*n* = 16) at different time points. Data are presented as median ± interquartile range. A significant difference at follow-up between the control group and the PBM group was observed using the Mann–Whitney *U* test, two-tailed (*P* = 0.035) PBM, photobiomodulation; CT, chemotherapy

At baseline, 6 weeks after initiation of chemotherapy, and at the end of therapy, no significant differences were observed between both groups in aerobic capacity and endurance using the 6MWT. However, at follow-up, a significantly higher score was observed in the PBM group (*P* = 0.035), as shown in Fig. 4. In addition, Table 2 shows a borderline significant difference in pain level at follow-up between both groups, with a higher score in the control group (*P* = 0.058).

**Table 2:** Patients’ pain level

	Control group (*n* = 16) Median (IQR)	PBM group (*n* = 16) Median (IQR)	*P*^a^
Baseline	0 (2)	0 (0.75)	0.634
Week 6 of CT	0 (1.75)	0 (2.75)	0.916
End of CT	1.5 (5.75)	1 (2.75)	0.754
Follow-up	1 (2.75)	0 (0.75)	**0.058**

Patients’ pain level using a numeric rating scale ranging from 0 (no pain) to 10 (worst pain imaginable). A borderline significant difference at follow-up between the control group (*n* = 16) and the PBM group (*n* = 16) was observed. *PBM*, photobiomodulation; *CT*, chemotherapy

Data in bold was set assuming a significance level of 5%

^a^Mann-Whitney *U* test, two-tailed

## Discussion

Results from the present NEUROLASER trial indicate that PBM has the potential to prevent the development of CIPN in breast cancer patients. Although not significant, the mTNS of the PBM group tended to be decreased compared with the mTNS of the control group. Patients experienced a better QoL in the PBM group over time compared to the control group. Furthermore, symptoms such as numbness in the hands, numbness in the feet, discomfort in the feet, and troubles feeling the shape of small objects in their hand deteriorated in the control group, whereas they remained constant in the PBM group.

The patient’s aerobic capacity and endurance in the PBM group were significantly better at follow-up than in the control group. Also, a lower pain score was observed at follow-up in the PBM group compared to the control group. These results indicate that PBM seems to expedite recovery of chemotherapy-induced side effects for breast cancer patients.

Only a limited amount of clinical trials was performed to investigate the use of PBM in patients affected with CIPN [29]. Argenta et al. conducted a randomized controlled trial with 68 patients with self-reported peripheral neuropathy and a history of taxane or platinum-based chemotherapy exposure. Several types of cancer were included. A significant reduction in mTNS was observed when treated 3-weekly for 6 weeks with PBM (class IV K-Laser, 800–970 nm, and 6.75–12 W) (*P* < 0.001) whereas it remained constant in the control group (*P* = 0.44) [26]. A prospective cohort study of Hsieh et al*.* with 17 gastrointestinal cancer patients treated with oxaliplatin-based chemotherapies showed similar results. After a 3-weekly PBM treatment (GaAlAs diode laser, 780 nm, 80 mW, and 48 J/cm^2^) for 4 weeks, a significant improvement in CIPN symptoms was observed using the Pain Quality Assessment Scale, Chemotherapy-Induced Neurotoxicity Questionnaire, Oxaliplatin-Specific Neurotoxicity Scale, touch-detection threshold, and cold-triggered pain withdrawal latency (*P*s < 0.05) [27]. Contrasting results between these trials and the NEUROLASER trial may be due to the curative versus preventive approach and the amount of PBM sessions. Furthermore, a variety of factors such as the type of chemotherapy, the chemotherapy dosage, non-blinded versus blinded scoring of CIPN, blinding of the patient during the procedure, and different outcome measures to assess CIPN could explain the different results.

One of the obstacles observed during the NEUROLASER trial was the assessment of CIPN. Although early identification is essential, a golden standard has not been established. Currently, the diagnosis of CIPN is mainly based on subjective descriptions by the patient. However, patients can be hesitant in reporting any symptoms in fear that their chemotherapy dose will be reduced, adversely affecting their treatment [30]. The literature describes two categories of CIPN grading tools: patient-reported outcome measurements and physician-based outcome measurements [33]. Patient-reported outcome measurements, such as FACT/GOG-Taxane and the European Organization of Research and Treatment of Cancer Quality of Life Questionnaire-CIPN twenty-item scale (EORTC-QLQ-CIPN20), have been validated for the detection of CIPN [34]. Physician-based outcome measurements, including (m)TNS, National Cancer Institute Common Terminology Criteria for Adverse Events (NCI-CTCAE), World Health Organization (WHO) criteria, and Eastern Cooperative Oncology Group (ECOG) scale are mainly based on the clinical examination. In general, these scales are organized in an ordinal fashion in which grade 0 represents the absence of symptoms and grade 4 or 5 represents the most severe symptoms. However, this type of assessment’s subjective nature often leads to a high degree of inter-observer variability [34, 35]. Nevertheless, the (m)TNS has proven to be a more objective and sensitive tool to assess CIPN than global screening tools [30, 36]. Another possibility to overcome the subjectivity in the assessment of CIPN is to perform nerve conduction studies (NCS). However, NCS are rarely conducted in the clinical oncological setting due to the need for specialized equipment and patients’ discomfort. Moreover, it mainly detects large myelinated fibers, whereas some chemotherapy drugs only affect small myelinated and unmyelinated fibers [2, 34]. To detect length-dependent small fiber neuropathy, skin punch biopsies at the distal thigh and distal leg can be taken to measure the loss of intra-epidermal nerve fibers [37]. However, this increases the risk for infection, especially in a population susceptible to neutropenia. Therefore, more research is needed to optimize CIPN assessment methods.

The safety of PBM and tumor cells needs to be monitored, especially because of the proliferative nature of this therapy. In addition, PBM was administered during chemotherapy. As a result, potential circulating cancer cells could be irradiated by PBM. A significant and growing literature indicates that PBM is safe and effective for use in supportive cancer care. However, some conflicting results are observed in in vitro studies investigating the effect of PBM on varied cancer cell lines. Yet, large differences exist in the PBM parameters and frequency of PBM applications within these studies [38]. Wikramanayake et al*.* investigated if PBM provided localized protection to cancer cells in a rat model. Shay chloroleukemic cells were injected subcutaneously before treating the rats with chemotherapy or PBM. Twenty percent of rats treated with cyclophosphamide remained leukemia-free, whereas 22% of rats treated with a combination of cyclophosphamide and PBM remained leukemia-free (*p* = 1.00), suggesting PBM did not compromise the efficacy of chemotherapy [39]. In addition, various clinical trials investigated the safety of PBM in patients with cancer. Most of them focused on the use of PBM for the management of oral mucositis during cancer therapy and observed no altered treatment outcomes [40–45].

Since this study was designed to be an initial exploration of the effects of PBM to prevent CIPN, it has some limitations that must be acknowledged. A major limitation is the small sample size. More than 70% of the eligible patients declined to participate in this trial. The main reason for this refusal is the additional demand that study protocol put on the patient during an already burdensome period. Furthermore, the COVID-19 pandemic slowed down the inclusion rate and resulted in seven drop-outs. Another limitation observed during this trial was the disbalance in exercise frequency between both groups at baseline. According to a recent study by Simsek et al., exercise can reduce the development of CIPN in breast cancer patients who received taxane treatment [46]. However, although patients in our trial allocated to the control group claimed to exercise more frequently at baseline (Table 1), this was not observed in the physical well-being subscale of the FACT/GOG-Taxane questionnaire (S2) nor the 6MWT (Fig. 4). In addition, our study also benefits from multiple strengths, including the prospective randomized design, the blinding of patients, well-defined PBM parameters, the preventive instead of curative approach, and the usage of validated grading tools and questionnaires.

## Conclusion

Despite the small sample size and need for more objective assessment tools with increased specificity and sensitivity, the NEUROLASER trial reported promising results concerning the prevention of CIPN with PBM in breast cancer patients. Symptoms such as numbness in the hands and feet deteriorated significantly in the control group, whereas they remained constant in the PBM group. In addition, a better patients’ QoL was observed in the PBM group. However, more randomized controlled trials with larger sample sizes and other cancer types are necessary to support these findings.

## Supplementary Information

Below is the link to the electronic supplementary material.

S1. Photobiomodulation parameters. MLS, Multiwave Locked System; CT, chemotherapy
Supplementary file1 (DOCX 16 kb)

S2. Functional Assessment of Cancer Therapy/Gynecologic Oncology Group Taxane (FACT/GOG-Taxane) subscales. Comparison of FACT/GOG-Taxane subscales between the control group (n=15) and the PBM group (n=16) at different time points. Data are presented as median ± interquartile range. A significant difference at follow-up between the control group and the PBM group was observed in the subscales physical well-being, emotional well-being, and functional well-being based on the Mann-Whitney test, two-tailed (Ps; 0.040). In addition, a significant difference between both groups was observed at baseline in the subscale functional well-being (P=0.044). PBM, photobiomodulation; CT, chemotherapy
Supplementary file2 (PDF 33 kb)

S3. Functional Assessment of Cancer Therapy/Gynecologic Oncology Group Taxane (FACT/GOG-Taxane) sensory peripheral neuropathy related questions. Based on the Friedman test, a significant difference over time was observed in the control group (n=15) in the scores of questions NTX1, NTX2, NTX4, and NTX9 (Ps 0.010), whereas they remained constant in the PBM group (n=16). Data are presented as a boxplot. PBM, photobiomodulation; CT, chemotherapy
Supplementary file3 (PDF 34 kb)

## Data Availability

Not applicable.
