# Supplementary material for: The use of photobiomodulation therapy for the prevention of chemotherapy-induced peripheral neuropathy: a randomized, placebo-controlled pilot trial (NEUROLASER trial)
Source: Support Care Cancer. 2022 Mar 21;30(6):5509–17. doi: 10.1007/s00520-022-06975-x (PMC8935622; doi:10.1007/s00520-022-06975-x)
Supplement: Supplementary file 1 — S1. Photobiomodulation parameters. MLS, Multiwave Locked System; CT, chemotherapy Supplementary file1 (DOCX 16 kb) [file 520_2022_6975_MOESM1_ESM.docx]

**Supplementary table 1.** Photobiomodulation parameters

| **Device information** | - Manufacturer - Model Identifier - Year Produced - Number of Emitters - Emitter Type - Beam Delivery System | - ASA srl - MLS® laser M6 - 2012 - 1 - 3 - Infrared laser diodes - Handpiece and scanning head | | |
| --- | --- | --- | --- | --- |
|  |  |  |  |  |
| **Irradiation parameters** |  | **Laser diode 1** |  | **Laser diode 2** |
|  | - Center wavelength | - 808 nm |  | - 905 nm |
|  | - Peak radiant power | - 1.1W |  | - 25W |
|  | - Beam divergence at 60% | - 42.8 mrad |  | - 59.2 mrad |
|  | - Maximum frequency   - Pulse on duration   - Duty cycle |  |  | - 90 kHz (1–2000 Hz)   - 100 ns single pulse width   - 50% |
|  | - Spectral bandwidth | ±5 nm | | |
|  | - Operating mode | Continuous pulsed wave mode | | |
|  | - Average radiant power | 3.3W | | |
|  | - Aperture diameter | 2 – 5 cm | | |
|  | - Irradiance at aperture | 0.168 W/cm2 | | |
|  | - Beam profile | Two laser beams work simultaneously and synchronously with coincident propagation axes | | |
|  |  |  | | |
| **Treatment parameters** | - Beam spot size at target area - Irradiance at target - Radiant exposure (fluence) - Number of points irradiated - Exposure duration - Application technique - Timing and frequency of treatment sessions | - 3.14 – 19.625 cm2 - 0.168 W/cm2 - 4 J/cm2 - Bilaterally at the upper limbs, the back, and lower limbs - 10–194 s - 5 cm above skin - Biweekly during taxane treatment (24 - 36 sessions in total depending on the CT regimen) | | |

MLS, Multiwave Locked System; CT, chemotherapy
