# Supplementary figures and images for: The use of photobiomodulation therapy for the prevention of chemotherapy-induced peripheral neuropathy: a randomized, placebo-controlled pilot trial (NEUROLASER trial)
Source: Support Care Cancer. 2022 Mar 21;30(6):5509–17. doi: 10.1007/s00520-022-06975-x (PMC8935622; doi:10.1007/s00520-022-06975-x)

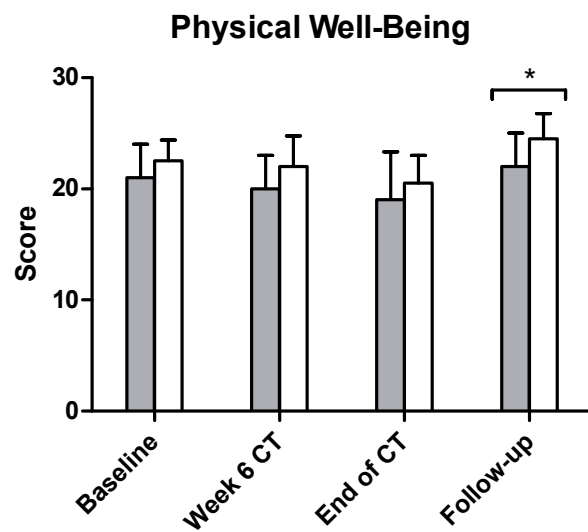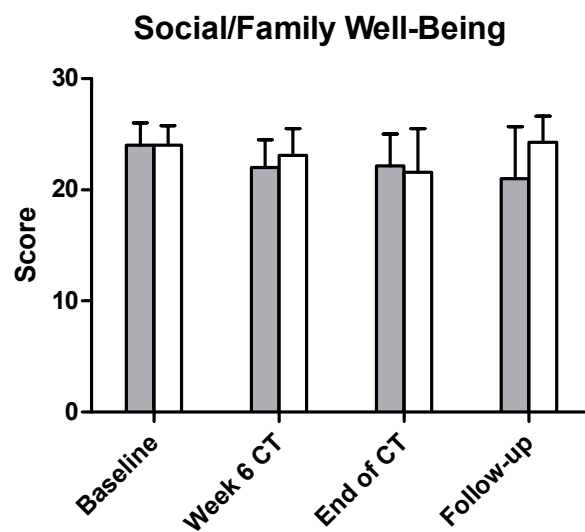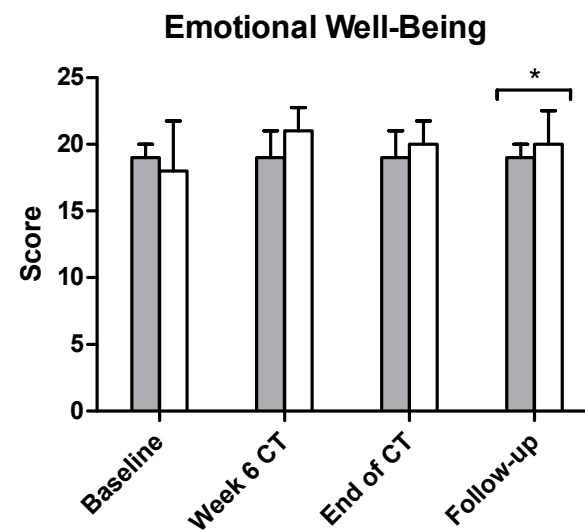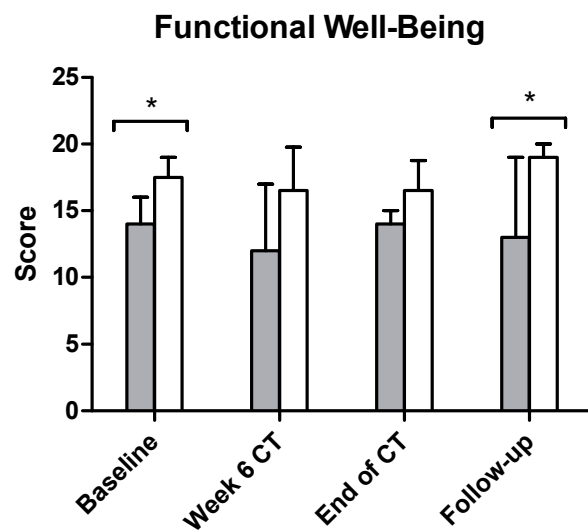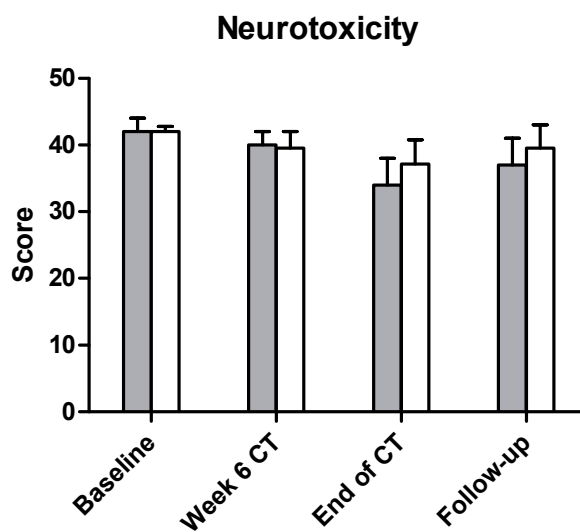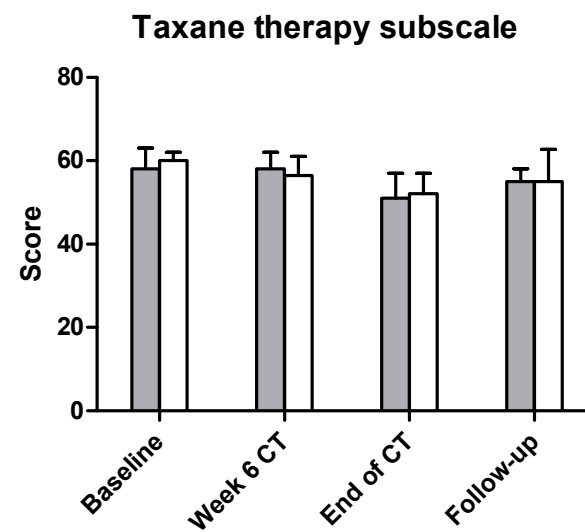

Supplement: Supplementary file 2 — S2. Functional Assessment of Cancer Therapy/Gynecologic Oncology Group Taxane (FACT/GOG-Taxane) subscales. Comparison of FACT/GOG-Taxane subscales between the control group (n=15) and the PBM group (n=16) at different time points. Data are presented as median ± interquartile range. A significant difference at follow-up between the control group and the PBM group was observed in the subscales physical well-being, emotional well-being, and functional well-being based on the Mann-Whitney test, two-tailed (Ps; 0.040). In addition, a significant difference between both groups was observed at baseline in the subscale functional well-being (P=0.044). PBM, photobiomodulation; CT, chemotherapy Supplementary file2 (PDF 33 kb) [file 520_2022_6975_MOESM2_ESM.pdf]

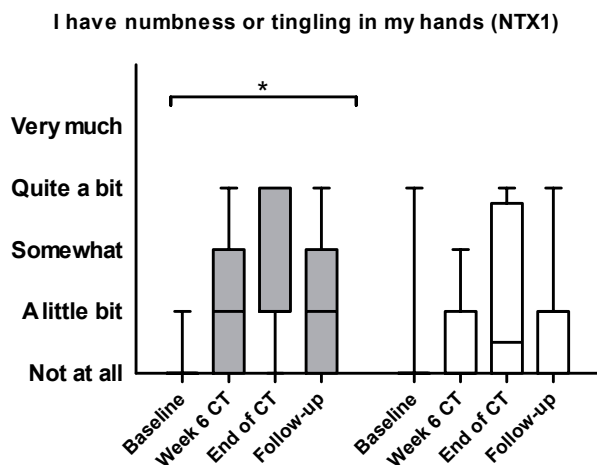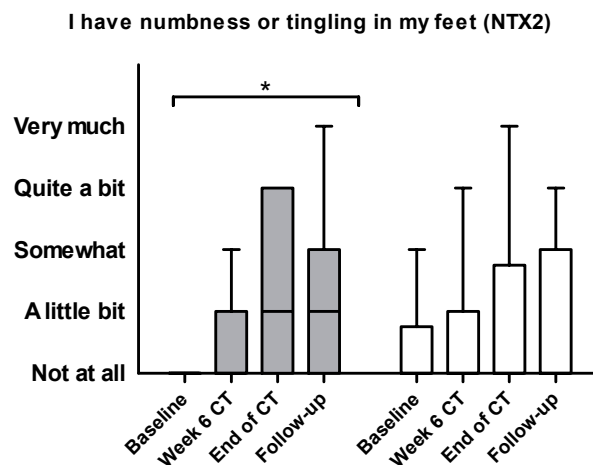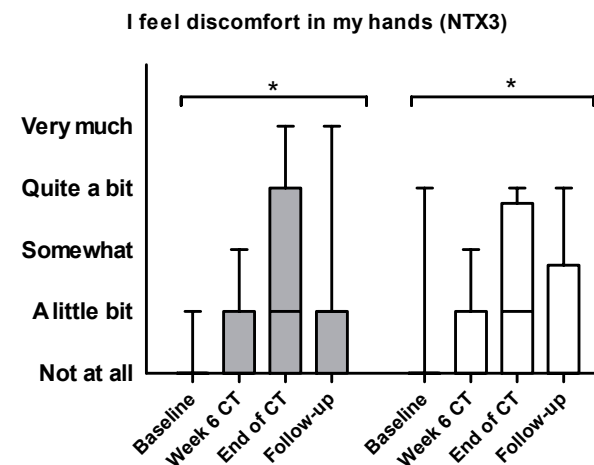

Control group  
PBM group

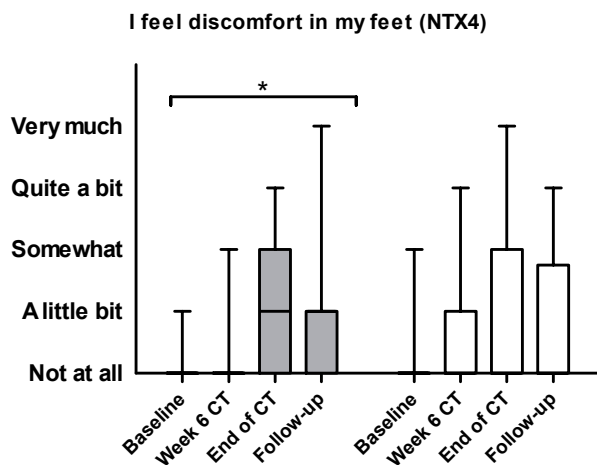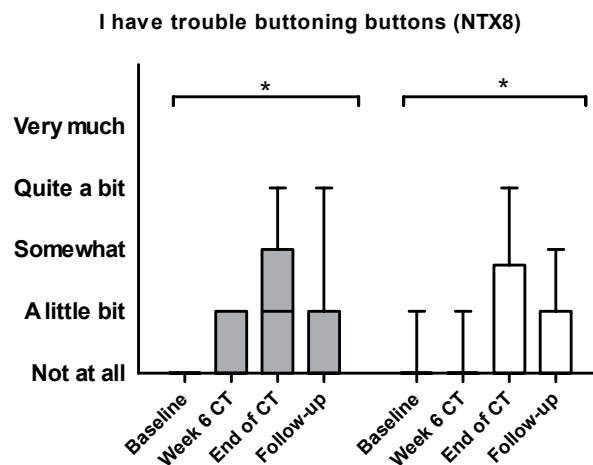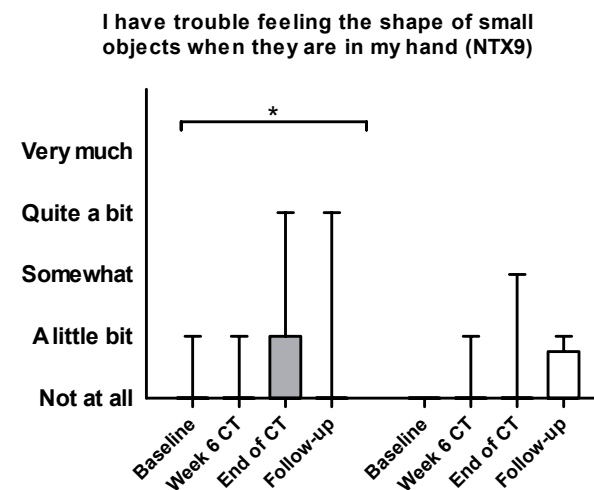

Supplement: Supplementary file 3 — S3. Functional Assessment of Cancer Therapy/Gynecologic Oncology Group Taxane (FACT/GOG-Taxane) sensory peripheral neuropathy related questions. Based on the Friedman test, a significant difference over time was observed in the control group (n=15) in the scores of questions NTX1, NTX2, NTX4, and NTX9 (Ps 0.010), whereas they remained constant in the PBM group (n=16). Data are presented as a boxplot. PBM, photobiomodulation; CT, chemotherapy Supplementary file3 (PDF 34 kb) [file 520_2022_6975_MOESM3_ESM.pdf]
